# Malignant Airway Obstruction and the Use of Nd:YAG Laser: A Systematic Review on Its Efficacy and Safety

**DOI:** 10.7759/cureus.34434

**Published:** 2023-01-31

**Authors:** Zaryab Umar, Muhammad Haseeb ul Rasool, Salman Ashfaq, Avish Parikh, Hannan A Maqsood, Asma U Hosna, Muhammad Ghallab, Nazaakat Ahmed, Jawad Khan, Jasmine K Sandhu, Jonathan Ariyaratnam, Theo Trandafirescu

**Affiliations:** 1 Internal Medicine, Icahn School of Medicine at Mount Sinai, Queens Hospital Center, New York, USA; 2 Medicine, Icahn School of Medicine at Mount Sinai, Queens Hospital Center, New York, USA; 3 Internal Medicine, Allama Iqbal Medical College, Lahore, PAK; 4 General Surgery, University of Michigan, Ann Arbor, USA; 5 Internal Medicine, Queens Hospital Center, New York, USA; 6 Medicine, Queens Hospital Center, New York, USA

**Keywords:** respiratory tract malignancy, laser treatment, nd:yag laser, airway stenosis, malignant airway obstruction

## Abstract

Endobronchial malignancies with significant airway obstruction can lead to multiple complications including pneumonia, and atelectasis over a period of time. Various intraluminal treatments have proven their value in palliative treatment for advanced malignancies. Nd:YAG (neodymium-doped yttrium aluminum garnet; Nd:Y3Al5O12) laser has established its role as a major palliative intervention due to its minimal side effects and improvement in quality of life by relieving local symptoms.

The systematic review was conducted with the goal of elucidating the patient characteristics, pre-treatment parameters, clinical outcomes, and possible complications resulting from the use of the Nd:YAG laser. A thorough literature search for relevant studies was conducted on PubMed, Embase, and Cochrane Library from the inception of the idea to November 24, 2022. Our study included all original studies including retrospective studies and prospective trials, but excluded case reports, case series with less than 10 patients, and studies with incomplete or irrelevant data. A total of 11 studies were included in the analysis. The primary outcomes focused on the evaluation of pulmonary functional tests, postprocedural stenosis, blood gas parameters after the procedure, and survival outcomes. Improvement in clinical status, improvement in objective scale for dyspnea, and complications were the secondary outcomes.

Our study shows that Nd:YAG laser treatment is an effective form of palliative treatment to provide subjective and objective improvement in patients with advanced and inoperable endobronchial malignancies. Due to the heterogeneous study populations in the studies reviewed and the presence of many limitations, more studies are still warranted to reach a definitive conclusion.

## Introduction and background

Endobronchial malignancy (and airway obstruction) is a common problem in small-cell lung cancer, non-small-cell lung cancer, and many other tumors that metastasize to the lungs from other parts of the body. The initial presentations of endobronchial malignancies are cough, hemoptysis, and dyspnea, which are distressing to the patients [1]. Among all lung cancer patients, 20-30% will develop complications, e.g., pneumonia, dyspnea, and atelectasis due to endotracheal and endobronchial diseases. Up to 40% of patients develop serious complications even after exhausting all treatment options including stenting, cryotherapy, mechanical dilation, and resection, photodynamic therapy (PDT), chemotherapy, and external beam radiotherapy [2,3]. Given the advancements in endoscopic treatment procedures along with their improved safety and effectiveness, laser treatment has become one of the most important endobronchial treatment procedures. Since the toxicity and side effects of chemotherapy and radiotherapy are very severe, patients seek alternative treatment options even if only for palliative purposes as they are well-tolerated with minimal side effects [4].

Nd:YAG (neodymium-doped yttrium aluminum garnet)-directed therapy for central airway obstruction (CAO) of the lungs has been a well-known treatment modality since the 1980s and has been well-established in the field [5]. It is most commonly used for palliative debulking procedures in metastatic obstructive diseases, where cardiothoracic surgery alone is not a suitable option or is not an option altogether. Initial data regarding the Nd:YAG laser was limited, partly due to the device's technical and mechanical limitations at the time. The 1064 nanometer (nm) wavelength available during the initial period significantly held back the data related to this topic as the laser was not able to provide sufficient cutting and coagulation capabilities to adequately perform resections. However, with the introduction of the 1318-nm wavelength, the data has shown improvement in symptoms in patients, and better survival rates in select patients [6].

Over the last few years, Nd:YAG laser has established itself as an important palliative treatment for patients with lung cancer [7]. Results from multiple studies have shown an improvement in quality of life and relieving local symptoms [8]. Treatment with Nd:YAG laser has led to significant improvement both objectively and subjectively in patients with endobronchial malignant obstruction. Interval response rate defined as tumor size at follow-up bronchoscopy performed at one week and one month was 53% and 23.5% in the Nd:YAG laser arm and 43% and 38.5% in the PDT arm, though the p-value was not significant [8]. The mean survival time after Nd:YAG laser treatment was 6.64 months [1]. It was also observed that the time for reintervention was longer in patients who received multimodal therapy where Nd:YAG laser was combined with either chemotherapy, radiotherapy, or brachytherapy than in those who received single modality treatment [1]. There was a significant improvement in the mean pulmonary function test after the procedure, which resulted in an improvement in the dyspnea index. Effective opening of the airway was achieved in 81% of the patients treated with Nd:YAG laser as compared to 75% of the patients who received photodynamic therapy (PDT) [9].

The aim of this article is to review current indications for and the role of endoscopic Nd:YAG laser therapy in the management of unresectable and advanced lung carcinoma, as well as to discuss overall survival rates and complications of the procedure.

## Review

Search strategy and study selection

This study followed Preferred Reporting Items for Systematic Reviews and Meta-Analyses (PRISMA) guidelines for systematic reviews and meta-analyses, which do not require protocol registration [10]. An electronic database search was conducted for relevant studies published from 11/20/2022 to 11/24/2022 on PubMed, Embase, and Cochrane using certain keywords. Table 1 provides a detail of the search terms used on PubMed, Cochrane, and Embase along with the results obtained.

**Table 1:** Search strategy implemented on the databases

Database	Search strategy	Results
PubMed	Bronchoscopy[tw] OR Bronch*[tw] OR Mesh terms: "Bronchoscopy"[Mesh] AND Nd:YAG[tw] OR “Nd:YAG laser”[tw] OR "Lasers"[Mesh] OR "Lasers, Solid-State"[Mesh] AND Airway[tw] OR Obstruction[tw] OR “Bronchial obstruction” [tw] OR “Tracheobronchial obstruction” [tw] OR "Airway Obstruction"[Mesh] AND “Lung cancer” [tw] OR Cancer[tw] OR Neoplasm[tw] OR Malig*[tw] OR Malignancy[tw] OR "Lung Neoplasms"[Mesh] OR "Neoplasms"[Mesh]	32
Cochrane	Bronchoscopy AND Laser OR Nd-YAG laser AND Tracheobronchial obstruction OR Bronchial obstruction OR Airway obstruction AND Lung neoplasm OR Lung malignancy OR Lung cancer OR Malignancy OR Tumor OR Cancer	1
Embase	Fiberoptic bronchoscopy OR Bronchoscopy AND Laser OR Neodymium YAG laser OR Neodymium Laser AND Airway obstruction OR Bronchus obstruction OR Trachea obstruction OR Trachea stenosis AND Lung cancer OR Lung tumor OR Neoplasm OR Malignant neoplasm OR Malignant	106

Our search included all original studies (cohort, cross-sectional, and case-control studies) describing the characteristics of patients with airway obstruction in the setting of malignancy, pre-treatment evaluation (including pulmonary function testing, scoring systems deployed to assess the severity of dyspnea, arterial blood gas parameters, imaging and degree of obstruction, etc.), outcomes and complications associated with the use of Nd:YAG laser, and commentaries and case series with more than or equal to eight patients. With respect to case series, emphasis was laid on including studies that provided sufficient data on the outcomes and variables pertinent to our study design. The exclusion criteria included non-original reports, which were either reviews, letters to editors, or commentaries that did not include patient data; case reports or case series involving fewer than 10 patients; unextractable or irrelevant data; articles not published in English; duplicate records; animal studies; overlapping data; and full texts that were not available, unextractable, or irrelevant data.

The primary outcomes that were the focus of our review were as follows: post-procedure stenosis, evaluation of pulmonary function testing, and blood gas parameters after the procedure. Survival outcomes were also the primary focus of our study. Secondary outcomes that we focused on in our systematic review were as follows: improvement in clinical status after laser treatment, and improvement in dyspnea grade/additional scoring systems and scales post-procedure. We also evaluated the complications of the procedure as a secondary outcome. To ensure that we did not miss any relevant studies, we manually searched the references of our included papers. All original studies that reported malignant airway obstruction and the use of Nd:YAG laser were included in the study. Our systematic review was screened by two independent reviewers for titles and abstracts, followed by a full-text screening to ensure that relevant papers were included. We resolved disagreements by discussion and by referring them to the senior author when necessary.

Data extraction

We developed a data extraction sheet using Microsoft Excel (Microsoft® Corp., Redmond, WA). Two independent reviewers extracted data using the Excel sheet. Disagreements and discrepancies were resolved through discussions with the senior author.

Quality assessment

The risk of bias in the included studies was evaluated by one independent reviewer. A risk-of-bias assessment tool developed by the National Institutes of Health (NIH) was used to assess the quality of the included studies [11].

Results

Search Results

A total of 139 records were identified. After the removal of two duplicate records, a total of 137 studies were included, which then underwent the screening process. After a thorough review of the study title and abstracts, 101 studies were excluded. It is noteworthy to mention that case reports were excluded during this phase.

**Figure 1:**
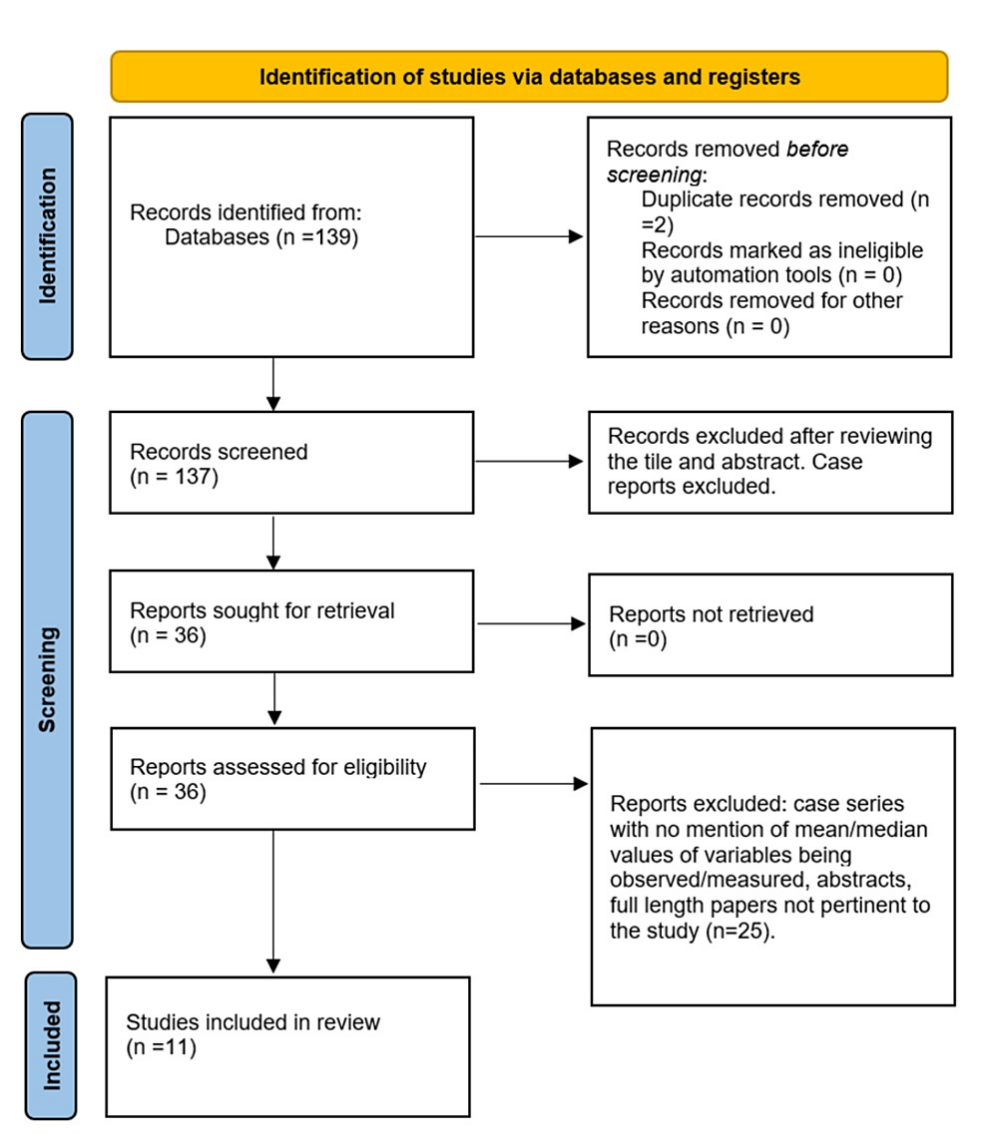
PRISMA flow diagram depicting the screening process for this systematic review and meta-analysis PRISMA: Preferred Reporting Items for Systematic Reviews and Meta-Analyses

A total of 36 reports became part of the secondary screening process. After the exclusion of case series with less than eight patients and no mention of mean/median values of variables pertinent to our study design, abstracts, and full-length papers not pertinent to the study, 11 studies were finally included in our systematic review (Figure 1).

Study Characteristics, Quality of the Included Studies, and Risk of Bias of Included Studies

These 11 studies included four retrospective studies [1,5,9,12], two prospective [7,13] and one retrospective case series [14], and four prospective studies (Table 2) [8,15-17]. Based on the NIH tool, all the studies included scored at least a 9 on the assessment. We evaluated the risk of bias of the case series included in our study using the NIH risk assessment tool.

**Table 2:** Baseline patient characteristics *Type and staging of malignancy (number of patients) in the PDT and Nd:YAG laser group: stage 1: 4 patients in total, 3 in the PDT and 1 in the laser group; stage 2: 1 patient in the PDT group; stage 3: 6 patients in total, 2 patients in the PDT and 4 in the laser group; stage 4: 10 patients in total, 3 in the PDT and 6 in the laser group. Recurrent tumor: 7 in total, 3 in the PDT group and 4 in the laser group; 25 patients with squamous cell carcinoma, 13 in the PDT and 12 in the laser group; 3 patients with adenocarcinoma, 2 in the PDT and 1 in the laser group; 3 patients with undifferentiated cancer in the laser group SD: standard deviation; T: tumor size; N: lymph nodal involvement

Authors	Study design	Year	Patient age	No. of patients (male, female, total)	Type of cancer/stage (number of patients)	Clinical presentation	Smoking status	Comorbid conditions
Han et al. [1]	Retrospective study	1999 and 2004	The age of the patient group ranged from 33 to 87 years, with a mean of 67 years	A total of 110 patients (78 male, 32 female)	Primary malignancy: non-small cell lung cancer (NSCLC) (80) (73%), squamous cell (50) (45%), adenocarcinoma (17) (15%), large cell (4) (4%), Unknown (9) (11%), small cell lung cancer (4) (3.5%), sarcoma (2) (2%), Secondary malignancy (20) (18%), Other (4) (3.5%). Staging of NSCLC: stage IB in 4% of diagnosed cases, stage II in 2% of diagnosed cases, stage III in 52% of the cases, and stage IV in 22% of the cases	Dyspnea, hemoptysis, and cough	Not mentioned	Not mentioned
Perin et al. [5]	Retrospective study	2012	Mean ± SD: 59 ± 9 years	464 patients (353 males and 111 females)	Squamous cell (326 patients), small cell (85), adenocarcinoma (16), carcinoid (6), metastases from lung primary (21), and other metastases (10). 64.6% had stage IV, 22.2% had stage IIIB, and 16.2% had stage IIIA disease	Not mentioned	Not mentioned	Acute myocardial infarction over 6 months before treatment (19 patients), arterial hypertension (20), chronic arrhythmia (9), chronic obstructive pulmonary disease (41), and stabilized cardiomyopathy (31)
George et al. [7]	Prospective case series	October 1987 and April 1989	The average age was 65 years, ranging from 40 to 80 years	55 patients (41 males and 14 females)	Squamous cell carcinoma (30), large cell carcinoma (11), adenocarcinoma (2), small cell carcinoma (6), adenoid cystic carcinoma (1), necrotic undefinable tumor, (1) and metastatic adenocarcinoma (4)	Dyspnea, cough, hemoptysis, malaise, weight loss, pneumonia, fever, sputum production, and paresis of recurrent nerve	Not mentioned	Not mentioned
Diaz-Jiménez et al. [8]	Prospective controlled trial	1999	Mean age ± SD: 65 ± 7 years	A total of 31 males were included in the study	Stage I (4), stage II (1), stage IIIA (6), stage IIIB (10), Stage IV (7), recurrent tumor (3), squamous cell cancer (25), adenocarcinoma (3), undifferentiated carcinoma (3)*	Dyspnea, cough, and hemoptysis	Not mentioned	Not mentioned
Furukawa et al. [9]	Retrospective study	1999	Mean age: 65 years (range: 41-84 years)	A total of 258 lesions were treated, 81 were treated via PDT, and 177 were treated via laser therapy. The PDT group had 78 cases (72 males and 6 females)	Squamous cell carcinoma (65), small cell carcinomas (4), adenocarcinoma (9), large cell carcinoma (2)	Severe dyspnea	Not mentioned	Not mentioned
Moghissi et al. [12]	Retrospective case series	1997	Mean age ± SD: 69 ± 9 years	17 patients (9 male and 8 females)	Squamous cell carcinoma (8), adenocarcinoma (5), small cell (1), large cell (1), adenoid cystic (1), and secondary from renal (1). The clinical stage of tumors was IIIa or IIIb (T3N2 or T4N1)	Not mentioned	Not mentioned	Renal malignancy, 1 patient
Gelb et al. [13]	Prospective case series	1990	Not mentioned	8 patients in total	Not mentioned	Cough, dyspnea, stridor, wheezing, or hemoptysis	Not mentioned	Not mentioned
George et al. [14]	Retrospective study	1987	Mean age: 65 years	97 patients [62 males (33 underwent laser treatment under local anesthesia and 29 under general anesthesia)]. [35 females (18 underwent treatment under local anesthesia and 17 under general anesthesia)]	Squamous cell carcinoma, 41 patients in the group requiring local anesthesia (L), and 26 patients in the group requiring general anesthesia (GA). Adenocarcinoma, L (2), 3 (GA). Large cell cancer, L (0), GA (1). Small cell cancer, L (0), GA (2). Adenoid cyst, L (3), GA (0). Carcinoid, L (1), GA (2). Malignant mixed, L (0), GA (1). Locally invasive thyroid cancer, L (1), GA (2). Poorly differentiated, L (0), GA (3). Metastatic, L (3), GA (6)	Hemoptysis and breathlessness	Not mentioned	Many of the patients had chronic hypoxic lung disease
Toty et al. [15]	Clinical trial	1981	Not mentioned	A total of 164 cases (24 primary untreated tracheal cancers, 48 recurrent cancers, 11 benign, 10 borderline malignant cancers, 47 iatrogenic stenosis patients, and 24 had granulomas)	Primary untreated tracheal cancers (24), recurrent cancers (48), amyloid tumor (1), histiocytoxanthofibroma (1), solitary papillomas (3), leiomyoma (1), diffuse papillomas (3), diffuse amylosis (2), carcinoids (7), cyclindromas (3)	Acute respiratory distress	Not mentioned	4 patients (respiratory, cardiac, or renal failure in elderly patients)
Gelb et al. [16]	Clinical investigation	1984	Partial obstruction group, mean age ± SD: 61 ± 11 years. Complete obstruction group, mean age ± SD: 66 ± 7 years	29 patients (18 men, 11 females). Partial obstruction group: 15 (11 men and 4 women). Complete obstruction group: 14 (7 men and 7 women)	Partial obstruction group: 10 had squamous cell carcinoma of the lung, 2 had adenocarcinoma of the lung, 1 had a large-cell undifferentiated carcinoma of the lung, and 2 patients had metastatic spread to the lung (one mucoepidermoid, one adrenal cell ca). Complete obstruction group: 6 squamous cell carcinoma, 7 large-cell undifferentiated lung cancer, and 1 hypernephroma	Uncontrolled cough, hemoptysis, dyspnea, or unresolved atelectasis, pneumonia	Not mentioned	Not mentioned
Moghissi et al. [17]	Prospective study	2006	Mean age: 68.5 years (range: 43-85)	1159 patients; 910 males and 249 females	Each patient had non-small cell lung cancer, stages IIIb and IV	Dyspnea, cough, and hemoptysis	355 patients were current smokers, 29 patients were nonsmokers, and 80 were former smokers	Not mentioned

Baseline Patient Characteristics

The baseline patient characteristics are summarized in Table 2. Toty et al., and the prospective case series by Gelb et al., do not make any mention of the age of the patients included in the studies [13,15]. Furukawa et al., Diaz-Jiménez et al., and the retrospective study and the prospective case series by George e al. report that the mean age of the patients was 65 years [7,9,14]. Han et al. and the retrospective and prospective study by Moghissi et al. and Perin et al. report the mean age of the patients included in their studies as follows: 67, 69, 68.5, and 59 years respectively [1,5,12,17]. The mean age of the patients in the partial obstruction group was 61 years and the mean age of the patients in the complete obstruction group was 66 years in the clinical investigation conducted by Gelb et al. [16].

The most common patient presentations were dyspnea, respiratory distress, cough, and hemoptysis. The prospective study by George et al. reports that some of the patients presented with malaise, weight loss, pneumonia, sputum production, and paresis of recurrent nerve [7]. The clinical investigation by Gelb et al. reports unresolved atelectasis and pneumonia as the presenting findings in a few patients [16]. The wide variety of malignancies reported by the studies is listed in Table 2.

A total of 258 lesions were treated, 81 via PDT and 177 treated via laser therapy, in the study conducted by Furukawa et al. [9]. The PDT group had 78 cases (72 were males and six were females). There was a total of 29 patients in the clinical investigation conducted by Gelb et al.; 15 patients had a partial obstruction and 14 patients had a complete obstruction [16]. Ninety-seven patients were included in the retrospective study conducted by George et al. (62 males, of which 33 underwent laser therapy under local anesthesia while 29 underwent laser therapy under general anesthesia; 35 were females: 18 were treated under local anesthesia and 17 under general anesthesia [14]. 

Pre-intervention Assessment and Intervention Details

Pre-intervention parameters are summarized in Table 3.

**Table 3:** Pre-intervention parameters WHO: World Health Organization; SD: standard deviation

Authors	Pulmonary function testing	Imaging	Site of lesion/location of the obstruction	Degree of obstruction	Dyspnea grade/additional scoring systems and scales used	Type of stenosis	Blood gas parameters
Han et al. [1]	Not mentioned	Not mentioned	Not mentioned	Not mentioned	Not mentioned	Not mentioned	Not mentioned
Perin et al. [5]	Not mentioned	Not mentioned	Trachea (95 patients), right main bronchus (180), left main bronchus (105), right upper lobe bronchus (48), and left upper lobe bronchus (36)	Not mentioned	Eastern Cooperative Oncology Group (ECOG score): inclusion criteria ≤3; exclusion criteria: ≥4	Intrinsic compression	Not mentioned
George et al. [7]	Not mentioned	Not mentioned	The trachea in six patients, the main carina in 8, a main bronchus in 27, and the right intermediate or lobar bronchus in 14 patients	Not mentioned	Not mentioned	Not mentioned	Not mentioned
Diaz-Jiménez et al. [8]	Not mentioned	Not mentioned	Right main bronchus in 12 cases, the left main bronchus in 8, the left superior bronchus in 5, the right inferior bronchus in 3, the intermediate bronchus in 2, and the left inferior bronchus in 1 case	Lesions caused >75% bronchial obstruction in 24 patients and <75% in the remaining 7	Patients with a Karnofsky performance status of more than or equal to 40% were included in the study. The Karnofsky performance status was performed before treatment and at 1 week and 1, 2, 3, 6, 12, and 18 months thereafter	Not mentioned	Not mentioned
Furukawa et al. [9]	Not mentioned	Not mentioned	Trachea and main bronchus: 26 lesions in the PDT group and 69 lesions in the laser group. Lobar and segmental bronchus: 55 lesions in the PDT and 108 in the laser group	Not mentioned	Patients had grade 2 (severe dyspnea) on the dyspnea scale	Patients had endoluminal stenosis leading to intrinsic compression	Not mentioned
Moghissi et al. [12]	Not mentioned	Chest X-ray	Tracheal bifurcation obstruction in 4 patients, 4 patients with left and 4 with right main bronchus obstruction, 2 patients with right intermediate bronchus obstruction, left lower lobe obstruction in 2 patients, left main bronchus and lower lobe obstruction in 1 patient	The overall degree of intraluminal tracheobronchial obstruction ranged from 60 to 100%	WHO performance status: range of 2-3 and mean of 2.24	Intrinsic (intraluminal) obstruction.	Not mentioned
Gelb et al. [13]	Forced expiratory volume in one second: 49% ± 12% of predicted. Forced vital capacity: 52% ± 18% of predicted	CT chest, chest X-ray.	3 patients had left main-stem bronchus lesion/obstruction; 2 patients had a right main stem and right upper lobe bronchus obstruction; 2 patients had right main-stem bronchus lesion/obstruction; 1 patient had left main-stem, right middle lobe, and right lower lobe bronchus lesion/obstruction	By direct bronchoscopy estimates, the mean main-stem bronchial diameter was 1.9 mm ± 1.6 mm (mean ± SD) before laser therapy	Not mentioned	Not mentioned	Not mentioned
George et al. [14]	The peak expiratory flow rate was 201 liters/min in patients undergoing local anesthesia and 185 liters/min in patients undergoing general anesthesia	Not mentioned	Proximal tracheobronchial tree	Partial obstruction (64 patients; 34 undergoing local anesthesia and 30 undergoing general anesthesia). Complete obstruction (10 patients; 4 undergoing local anesthesia and 6 undergoing general anesthesia)	Karnofsky performance index [Mean (SD)]: 66 (18) for patients undergoing local and 64 (23) for patients undergoing general anesthesia	Intrinsic compression (intraluminal obstruction). 1 patient had fistula formation in the setting of malignancy (undergoing general anesthesia)	Not mentioned
Toty et al. [15]	Not mentioned	Not mentioned	Trachea or the carina, on a bronchial suture line, or in one main bronchus	Not mentioned	Not mentioned	Not mentioned	Not mentioned
Gelb et al. [16]	Not mentioned	Not mentioned	Not mentioned	Not mentioned	Partial obstruction: dyspnea index (mean ± SD), before laser treatment: 3.4 ± 0.6. Complete obstruction: dyspnea index, before laser: 3.6 ± 0.5	Not mentioned	Not mentioned
Moghissi et al. [17]	Not mentioned	Chest X-ray	Trachea, carina, and main stem bronchus	Malignant endobronchial tumor obstruction ranging from 50-95% (mean: 75%)	Not mentioned	Intrinsic compression	Not mentioned

In the prospective case series conducted by Gelb et al., the mean forced expiratory volume in one second (FEV1) was 49 ± 12% of predicted, and forced vital capacity (FVC) of 52% ± 18% of predicted [13]. In the retrospective study by George et al., the average peak expiratory flow rate (PEFR) was 201 liters/minute in patients undergoing laser therapy under local anesthesia and 185 liters/minute in patients undergoing laser therapy under general anesthesia [14]. The sites/locations of the obstruction in the setting of malignant lesions have been described in Table 3.

The malignant lesions caused more than 75% bronchial obstruction in 24 patients and less than 75% obstruction in the remaining seven patients in the study by Diaz-Jiménez et al. [8]. The prospective case series by Gelb et al. noted the mean main-stem bronchial diameter to be 1.9 mm ± 1.6 mm before laser therapy [13]. The retrospective study by George et al. observed partial obstruction in 64 patients and complete obstruction in 10 patients [14]. The retrospective case series by Moghissi et al. reported the overall degree of intraluminal tracheobronchial obstruction ranging from 60-100% [12]. Malignant endobronchial tumor obstruction ranged from 50-95% (mean 75%) in the prospective study conducted by Moghissi et al. [17].

Furukawa et al. observed that the patients had grade 2 (severe dyspnea) on the dyspnea scale [9]. Patients with a Karnofsky performance status of more than or equal to 40% were included in the study by Diaz-Jiménez et al. The Karnofsky performance status assessment was performed before treatment initiation, at one week, and one, two, three, six, 12, and 18 months thereafter [8]. The clinical investigation by Gelb et al. observed the average dyspnea index before laser treatment to be 3.4 ± 0.6 in patients with partial obstruction and 3.6 ± 0.5 in patients with complete obstruction [16]. The retrospective study by George et al. noted the mean Karnofsky performance index for patients undergoing local anesthesia for laser treatment to be 66 and 64 for patients undergoing general anesthesia for laser treatment [14]. The WHO performance status in patients in the retrospective case series by Moghissi et al. ranged from 2 to 3 with a mean of 2.24 [12]. Patients with an Eastern Cooperative Oncology Group (ECOG) ≤3 were included in the study by Perin et al. [5]

The intervention details are described in Table 4. Toilet bronchoscopy was performed in all patients after Nd:YAG laser treatment in the study by Furukawa et al [9]. Toty et al. reported 48 recurrent cases of cancer undergoing radiotherapy prior to laser treatment. Some of the recurrent cancer patients underwent chemotherapy before the laser treatment; 18 patients with recurrent cancer had prior surgical intervention (six had pneumonectomies) [15]. Three patients in the PDT group received external radiotherapy prior to laser therapy in the study conducted by Diaz-Jiménez et al [8]. Seven patients underwent chemotherapy, 50 patients underwent radiotherapy, and 17 patients underwent brachytherapy prior to Nd:YAG laser therapy in the study performed by Han et al. [1]. The clinical investigation by Gelb et al. reported 25 patients being previously treated with a combination of surgical intervention, radiation, and chemotherapy, whereas 39 patients underwent either surgical intervention, chemo, or radiotherapy prior to laser treatment in the retrospective study by George et al. [14,16]. The retrospective case series by Moghissi et al. noted three patients undergoing chemotherapy, eight patients undergoing radiotherapy, and eight patients undergoing mechanical debulking prior to laser intervention. Nd:YAG laser therapy was followed by PDT therapy in all the patients in the study [12]. Of note, 80% of the patients included in the prospective study by Moghissi et al. underwent other cancer therapy prior to the laser intervention. Patients with endobronchial recurrence of the tumor status post-Nd:YAG laser were offered repeat treatment. Also, 17 patients with locally advanced bulky tumors with no evidence of metastatic disease underwent PDT four to six weeks after laser therapy [17]. Perin et al. reported 329 patients receiving chemotherapy, 76 patients receiving external beam radiotherapy, and 127 patients receiving some sort of interventional pulmonology treatment prior to Nd:YAG laser therapy [5]. 

**Table 4:** Interventions SD: standard deviation; N/A: not applicable

Authors	Details of bronchoscope used for laser treatment	Treatment prior to laser therapy	Details of Nd:YAG laser used	Number of treatments	Post-laser therapy
Han et al. [1]	Procedures were performed under general anesthesia using a rigid bronchoscope and/or fiberoptic bronchoscope	Chemotherapy in 7 and radiotherapy in 50 patients. Brachytherapy in 17 patients	Laser treatments were delivered using an Nd:YAG laser. A series of pulses between 0.5 and 1.0 seconds was used, with a power setting of 12 to 30 watts	87 patients received one laser treatment only. 23 patients received multiple treatments, accounting for 66 laser interventions in total, with one patient receiving 9 treatments	N/A
Perin et al. [5]	Laser therapy was carried out by a combination of rigid and flexible bronchoscopies performed	329 patients had previous chemotherapy. 76 patients had previous external beam radiotherapy. 127 patients had previous interventional pulmonology treatment	Sharplan-3000 Nd:YAG laser machine (Laser Industries Ltd., Or Akiva, Israel). The average power used was 50-60 watts, with 1-2-second pulses. The average duration of procedures was 60 minutes	Details on the number of treatments given were not mentioned	N/A
George et al. [7]	Patients were initially assessed by fiberoptic bronchoscopy	N/A	All patients were treated with the 1.32-micrometer output beam of a specially designed continuous wave Nd:YAG laser (Medilas 2; MBB Medizintechnic). The power output of the laser ranged from 2 to 30 watts and the pulse duration from 0.1 to 9.9 seconds	A total of 106 laser treatments were given to 55 patients, with 12 receiving more than one course for recurrent airway obstruction	N/A
Diaz-Jiménez et al. [8]	Rigid bronchoscopy for every patient undergoing laser treatment	Three patients in the PDT group received external radiotherapy	Nd:YAG resection was performed using 15 ± 80 watts pulses and a pulse duration of 0.5 ± 1.5 seconds	Five patients in the PDT group required a second dose of dihematoporphyrin-ether (DHE) 1 (n=2), 3 (n=1), 4 months (n=1), and 1 year (n=1) following the first treatment. Only one patient in the Nd:YAG laser therapy group required a second laser photo resection, 45 days after the first treatment	N/A
Furukawa et al. [9]	Flexible bronchoscopy used for both laser and photodynamic therapy	N/A	Nd:YAG laser system (Olympus, model MYL-2). The energy was from 20 to 40 watts, and the wavelength at 1,064 nm was irradiated to the lesions. Total irradiation energy was 200 J on average (total laser energy of Nd:YAG laser was 12,500 J at 30 watts). Laser irradiation time was 1.0 to 2.0 seconds repeatedly	Laser irradiation of Nd:YAG laser was necessary more than twice (69%) and used once in 31% of the lesions being treated with laser treatment. PDT treatment was necessary once in 91% of the lesions and twice in 9% of the lesions	Toilet bronchoscopy post laser
Moghissi et al. [12]	Moghissi-Jessop bronchoscope	3 patients underwent chemotherapy, 8 patients underwent radiotherapy, and 8 patients underwent mechanical debulking prior to laser therapy	Power of 40-60 watts in pulses of 4 seconds	Results were recorded after one treatment cycle comprising YAG laser followed by PDT was completed	Nd:YAG laser therapy was followed by endoscopic PDT. PDT was given 4-6 weeks later. Patients were intravenously injected with polyhematoporphyrin, 2 mg/kg body weight, and 24-48 hours later they received PDT with illumination by 630 nm light generated by a copper vapor (Oxford) laser
Gelb et al. [13]	Fiberoptic bronchoscopy	N/A	Not mentioned	Details on the number of treatments given were not mentioned	N/A
George et al. [14]	Bronchoscopy was performed with an Olympus BF lTIO fiberoptic instrument	39 patients (local anesthesia group) and 30 patients (general anesthesia group) received either surgery, radiotherapy, or chemotherapy	Fibrelase 100 (Pilkington Medical Systems Ltd, Scotland). Power of 70 watts (local anesthesia group) and 50-70 watts (general anesthesia group), given in pulses lasting no longer than 2 seconds. The mean (SD) duration was 147 (164) days for local anesthesia and 79 (54) days for the general anesthesia group	Responders: 27 patients received 35 courses of treatment (local anesthesia group; 1.97 treatment sessions per course) and 31 patients received 57 courses (general anesthesia; 1 treatment session per course); the difference was highly significant (p<0.001). Non-responders: the 24 patients who did not respond to treatment under local anesthesia received an average of 1.75 treatment sessions. In each case, treatment was continued until all treatable tumors had been resected and cauterized. With general anesthesia, treatment could be completed in one session in all but one of the 15 non-responders. The overall difference between the two groups was significant at the 5% level	N/A
Toty et al. [15]	Standard rigid bronchoscope and a fiberscope	Some of the recurrent cancer patients underwent chemotherapy. 48 recurrent cases of cancer underwent radiotherapy. 18 patients with recurrent cancer had prior surgical intervention (6 had pneumonectomies)	Compagnie Industrielle des Lasers (CILAS). Power of 50 to 90 watts. 30 watts for 0.7 to 1 second. 40 to 90 watts for 0.3 seconds in bursts of one every two seconds	95 out 0f 164 patients had one session, the rest between two and five	N/A
Gelb et al. [16]	Flexible fiberoptic bronchoscope	25 of the patients had been previously treated with combination(s) of surgical procedure, radiation therapy, and chemotherapy	Power of 40 to 60 W with an individual pulse time of 0.4 to 0.7 seconds in bursts every two seconds, with a target distance of 5 to 10 mm	A total of 45 laser treatments in 29 patients (18 men) aged 39 to 82 years, all of whom had biopsy-proved malignancy	N/A
Moghissi et al. [17]	A purpose-designed rigid bronchoscope (Moghissi-Jessop bronchoscope)	Over 80% of the patients underwent other cancer therapy, predominantly external beam radiotherapy, prior to Nd:YAG laser treatment	The laser equipment initially consisted of a Fibrelase 100 (Pilkington Medical Systems Ltd, Scotland) and then a Sharplan 3000 laser (Sharplan Lasers Inc). The laser power density used was 20 to 50 watts, delivered in pulses of 2 to 10 seconds via an optical fiber, in non-contact mode. Patients received treatment for a total of 4-6 weeks	A total of 1159 patients received 2235 treatments	Patients with evidence of endobronchial recurrence of tumor were offered repeat Nd:YAG laser or other appropriate treatment. A small group of 17 patients, with locally advanced bulky tumors in the main stem bronchus extending to the lower trachea and carina, but clinically without metastatic disease, had bronchoscopy PDT 4-6 weeks following Nd:YAG laser treatment, as per design of the prospective study

Outcomes

The primary and secondary outcomes that were the focus of our study are summarized in Table 5.

**Table 5:** Outcomes and complications *Significant difference before and after laser treatment (p<0.05). ^Significant difference between the partial obstruction group and the complete obstruction group (p<0.05) SD: standard deviation; FEV1: forced expiratory volume in 1 second; FVC: forced vital capacity

Authors	Post-procedure stenosis	Post-procedure pulmonary function testing	Post-procedure dyspnea grade/additional scoring systems and scales used	Post-procedure blood gas parameters	Survival outcomes	Other	Complications
Han et al. [1]	Not mentioned	Not mentioned	Not mentioned	Not mentioned	Median survival after Nd:YAG laser treatment was 6.64 months; median survival in patients who received single modality treatment was 3.79 months; median survival in patients who received multimodality treatment was 6.99 months	After Nd:YAG laser intervention, 76% of patients reported improvement in dyspnea, 94% for hemoptysis, and 75% for cough. 55% of patients were rendered asymptomatic. In 23 patients who had multiple laser interventions, the length of time to reintervention after single-modality laser intervention alone was compared with the length of time to reintervention after multimodal treatment. Of the 66 treatments, 37 were single modality and 29 were multimodality. The median time to reintervention in patients who received multimodal treatment in addition to laser treatment (3.9 months) was significantly longer than that for patients who received laser treatment alone (median 2.2 months)	2 patients had pneumonia. 10 patients died within 30 days (the 30-day mortality was 6.5%)
Perin et al. [5]	Not mentioned	Not mentioned	Not mentioned	Not mentioned	Not mentioned	Not mentioned	Pneumothorax: 4 patients. Global respiratory failure: 3 patients. Bleeding: 12 patients. Death: 1 patient. Tracheal-esophageal fistula: 1 patient Arrhythmia: 2 patients. Prolonged hypoxemia: 11 patients. Pneumomediastinum: 1 patient
George et al. [7]	Partial obstruction (number of patients): trachea/carina (14): endoscopic improvement in 14 patients (100%). Main bronchus (16): endoscopic improvement in 13 patients (81%). Interlobar/lobar bronchus (14): endoscopic improvement in 9 patients (64%). Complete obstruction (number of patients): main bronchus (11): endoscopic improvement in 10 patients (84%)	A 20% increase in FVC was seen in 5 out of 8 (62%) patients with main bronchial obstruction and in 3 out of 8 (38%) patients with intermediate/lobar bronchial obstruction	Not mentioned	Not mentioned	Survival from the time of the first laser treatment was reasonably durable with 50% of patients remaining alive at 7 months. The overall morbidity was 6.6% of all treatments (12.7% of all patients), while the postoperative mortality was 1.8% of all treatments (3.6% of all patients)	Partial obstruction: trachea/carina (14): symptomatic improvement in 14 patients (100%). Main bronchus (16): symptomatic improvement in 13 patients (81%). Interlobar/lobar bronchus (14): symptomatic improvement in 9 patients (64%). Complete obstruction: main bronchus (11): symptomatic improvement in 10 patients (84%). Objective response (number of patients): partial obstruction: trachea/carina (14), 14 patients, 100%. Main bronchus (16), 5 patients, 62%. Interlobar/lobar bronchus (14), 3 patients, 38%. Complete obstruction: main bronchus (11), 32 patients, 80%	Pneumonia: 1 patient. Pneumothorax: 1 patient. Respiratory distress: 2 patients. Bleeding: 5 patients. Death: 43 patients
Diaz-Jiménez et al. [8]	Airway obstruction improved from 79% to 62% in 27 patients, overall	Not mentioned	Not mentioned	Not mentioned	Not mentioned	At the 1-week follow-up examination, the response rate was 43% in the PDT group versus 53% in the Nd:YAG laser resection group; the corresponding figures at 1 month were 38.5% versus 23.5%, (p=NS). The amelioration of symptoms was similar in both groups. Dyspnea, hemoptysis, cough, and sputum production improved within 1 week after treatment. Between 1 week and 1 month post-treatment, dyspnea, hemoptysis, and sputum production showed greater improvement than did cough	Bronchitis: 4 patients in the PDT group and 1 patient in the laser group. Photosensitization in four patients in the PDT group and dyspnea in three patients (both groups combined). Two patients (one in each group) died from massive hemoptysis and presumed progression of the disease. By the end of the study, 23 (74.2%) of the 31 patients had died
Furukawa et al. [9]	Effective opening in 143 of 177 lesions (81%) treated via Nd:YAG laser. 93% effective opening (64 of 69 lesions) in the trachea and main bronchus while 73% effective opening (79 of 108 lesions) in the lobar and segmental bronchus after laser treatment. Effective opening in 61 of 81 lesions treated by PDT therapy (75%), 73% effective opening (19 of 26 lesions) in the trachea and main bronchus while 76% (42 of 55 lesions) in the lobar and segmental bronchus after PDT treatment	Not mentioned	Not mentioned	Not mentioned	Not mentioned	N/A	2 patients in the PDT group experienced fever. 12 patients in the laser treatment group suffered from pneumonia while 4 patients in the PDT group suffered from the disease. Bleeding was a complication seen in 10 patients undergoing laser therapy. Arrhythmia in 8 patients and airway rupture in 4 patients undergoing laser treatment. Death was a complication seen in 1.7% of the patients undergoing laser therapy
Moghissi et al. [12]	Mean percentage increase in luminal opening: 66% (range: 40-90%)	The mean improvement in FEV1 was 25% (range: 0-70%). The mean improvement in FVC was 28% (range: 0-90%)	Not mentioned	Not mentioned	11 of the 17 patients (65%) survived for 1 year and 8 (47%) for 2 years; the median survival of the 10 patients who had died by the time of writing the study was 18.5 months (range: 5-39)	Re-expansion on chest X-ray	Skin photosensitization: 1 patient
Gelb et al. [13]	By direct bronchoscopy estimates, the mean main-stem bronchial diameter was 1.9 mm ± 1.6 mm before laser therapy; after laser therapy, the mean diameter increased to 9.6 mm ± 1.0 mm	Not mentioned	Not mentioned	Not mentioned	Not mentioned	Not mentioned	Not mentioned
George et al. [14]	Not mentioned	Mean (SD) increases in PEFR were 86% (92%) for the local anesthesia group and 114% (115%) for the general anesthesia group	Not mentioned	Not mentioned	Not mentioned	27 patients (19 with partial obstructions, 1 with complete obstruction, and 7 presenting with hemoptysis) out of 51 patients receiving local anesthesia showed an objective response. 31 patients (22 with partial obstructions, 2 with complete obstruction,s and 7 presenting with hemoptysis) out of 46 patients receiving general anesthesia showed an objective response	Pneumonia: 1 patient each in the local and general anesthesia group. Respiratory distress: 1 patient in the general anesthesia group. Bleeding/hemorrhage: 2 patients in the local anesthesia group. Death: 4 patients in the local anesthesia and 1 patient in the general anesthesia group. Myocardial infarction: 3 patients in the local anesthesia and 1 patient in the general anesthesia group. Tracheal-esophageal fistula in 1 patient from the general anesthesia group. Arrhythmia: 2 patients in the general anesthesia group. Cerebrovascular accident: 1 patient undergoing general anesthesia (with etomidate use)
Toty et al. [15]	Not mentioned	Not mentioned	Not mentioned	Not mentioned	Not mentioned	All patients with recently diagnosed cancer noted significant improvement in respiratory status following treatment. 9 patients with recurrent cancer remained alive 1 year after the treatment. 1 recurrence in moderately malignant tumors after treatment	Fever: 1 patient. Death: 1 patient
Gelb et al. [16]	Not mentioned	Partial obstruction: FEV 1 (% predicted mean ± SD) before laser (56.0 ± 22.0). After laser (67.0 ± 27.0). Complete obstruction: FEV 1 % predicted mean ± SD) before laser (46.0 ± 14.0). After laser (48.0 ± 15.0). Partial obstruction: FVC (% predicted mean ± SD) before laser (68.0 ± 21.0). After laser (76.0 ± 24.0). Complete obstruction: FVC (% predicted mean ± SD) before laser (47.0 ± 16). After laser (49.0 ± 17.0). Partial obstruction: Mid expiratory flow rate (MMF) (% predicted mean ± SD) before laser (27.0 ± 14.0). After laser (62.0 ± 24.0). Complete obstruction: MMF (% predicted mean ± SD) before laser (33.0 ± 12.0). After laser (50.0 ± 9.0)*	Partial obstruction: dyspnea index (mean ± SD) before laser (3.4 ± 0.6). After laser (2.6 ± 0.6)*. Complete obstruction: dyspnea index (mean ± SD) before laser (3.6 ± 0.5). After laser (3.4 ± 0.5). Partial obstruction: Karnofsky (% mean ± SD) before laser (45.0 ± 15.0). After laser (60.0 ± 15.0)*. Complete obstruction: Karnofsky (% mean ± SD) before laser (28.0 ± 11). After laser (29.0 ± 16.0)	Not mentioned	Not mentioned	The overall success that lasted 1 to 6 months: 13 patients with partial obstruction*. 5 patients with complete obstruction^	Respiratory failure: 3 patients. Bleeding: 2 patients
Moghissi et al. [17]	Mean 48% increase	Mean 15% increase in FEV1 (range 10-28%). Mean 27% increase in FVC (range 25-35%).	Not mentioned	Not mentioned	A study of 17 patients showed 47% 2-year survival	Chest X-ray showed improvement in all. Symptomatic relief from dyspnea was moderate to very much	Pneumonia: 3 patients. Respiratory distress: 10 patients. 36 patients encountered non-fatal hemorrhages and 1 fatal hemorrhage. Death: 2 patients. Tracheal stenosis: 1 patient. Fatal myocardial infarction: 1 patient

Furukawa et al. reported effective opening in 81% of the patients treated with Nd:YAG laser and 75% of the patients treated with PDT [9]. Toty et al. noted that all patients had improvement in their clinical status following laser therapy. Nine patients with recurrent cancer remained alive one year after the treatment. One patient with a moderately malignant tumor had a recurrence following laser therapy [15].

Airway obstruction improved from 79% to 62% in 27 patients post-therapy in the study conducted by Diaz-Jiménez et al. (both laser and PDT groups combined). At the one-week follow-up, the response rate (tumor size at follow-up bronchoscopy) was 53% in the patients who received laser treatment versus 43% in the patients who received PDT. The corresponding figures at one month were 23.5% and 38.5% respectively; however, the p-value was non-significant. In both groups, improvement in symptoms appeared to be similar [8].

As per the prospective study conducted by George et al., patients with both partial and complete obstruction showed symptomatic improvement and an improvement in the degree of stenosis following laser treatment. A 20% increase in FVC was seen in five out of eight (62%) patients with main bronchial obstruction and in three out of eight (38%) patients with intermediate/lobar bronchial obstruction; 50% of patients remained alive seven months after treatment. The overall morbidity was 6.6% of all treatments (12.7% of all patients), while the postoperative mortality was 1.8% of all treatments (3.6% of all patients) [7]. Han et al. reported a median survival of 6.64 months after Nd:YAG laser treatment. Patients were found to have an improvement in dyspnea, cough, and hemoptysis status post-treatment; 55% of the patients were rendered asymptomatic. The study also mentions that the time to reintervene after multimodal treatment (Nd:YAG laser combined with either chemotherapy, radiotherapy, chemoradiotherapy, or brachytherapy) was longer than compared to the use of a single modality (Nd:YAG laser only) for the treatment of airway obstruction in the setting of malignancy [1].

The mean main-stem bronchial diameter improved from 1.9 ± 1.6 mm to 9.6 ± 1.0 mm in the participants of a prospective case series conducted by Gelb et al. [13]. The clinical investigation by Gelb et al. reported that the mean FEV1, FVC, and mid-expiratory flow rate (MMF) improved in patients with both partial and complete obstruction. Post-laser therapy, the dyspnea index was significantly better than the initial dyspnea index (p<0.05) and was associated with significant improvement in the Karnofsky score in patients with partial obstruction. The dyspnea index and the Karnofsky score did improve in patients with complete obstruction. The Karnofsky score and FVC were significant (p>0.05) when compared to patients with complete obstruction [16].

The retrospective study by George et al. noted a mean increase in PEFR of 86% in patients who received treatment under local anesthesia and 114% in patients who received treatment under general anesthesia; 27 out of 51 patients who received treatment under local anesthesia showed objective response whereas 31 out of 46 patients who received treatment under general anesthesia showed an objective response [14].

The mean improvement in FEV1 and FVC was noted to be 25% and 28% respectively among patients receiving Nd:YAG laser treatment, as demonstrated by the retrospective case series conducted by Moghissi et al. The median survival of the 10 patients who had died by the time of writing the study was 18.5 months (range: 5-39) [12]. A mean 15% increase and 27% increase in FEV1 and FVC were observed after laser treatment in the prospective study by Moghissi et al. [17]. A study of 17 patients showed a two-year survival of 47%. Unfortunately, Perin et al. did not report outcomes pertinent to our study apart from the complications of the laser treatment [5].

Discussion

The use of Nd:YAG laser treatment in patients with luminal obstruction of the trachea-bronchial respiratory tract due to malignancy has demonstrated positive outcomes in the form of subjective and objective improvement, regardless of the age group, type of malignancy, brand of laser, degree or location of the obstruction, or use of other modalities in conjunction with the laser treatment. The effectiveness of the treatment is largely dependent on complete resection of the lesion, if possible. As reported by Rolle A. et al., the prognostic capability of the traditional risk factors, such as solitary vs. multiple lesions, disease-free interval, and the number of metastases present, diminished in patients in whom complete resection was performed using the Nd:YAG laser. They found that 36% of the patients with four or more metastatic lesions were able to achieve confirmed five-year survival, whereas 28% of patients with 10 or more metastases, and 26% of patients with 20 or more metastatic lesions were able to achieve five-year survival, further elaborating the point that complete resection is arguably the most important prognosticating factor [6]. Another important factor that has impacted the effectiveness of this method is the tumor size and location; central, small tumors had more favorable outcomes than large, segmental tumors. Better outcomes have also been noted when using a higher wavelength of the laser instead of the conventional 1064-nm wavelength [7,9].

Multiple outcomes were used to assess objective improvement in the study conducted by Han et al. Patients experienced a subjective improvement in the form of an improvement in the presenting symptoms of dyspnea, hemoptysis, and cough, with some patients experiencing complete resolution of symptoms (after Nd:YAG laser intervention, 76% of patients reported improvement in dyspnea, 94% for hemoptysis, and 75% for cough; 55% of patients were rendered asymptomatic) [1]. 

The outcomes noted in our studies were mostly favorable, showing better responses as compared to PDT, as demonstrated by Diaz-Jiménez et al., and marked symptomatic improvement in patients after the intervention, as noted by George et al. and Han et al [1,7,8]. Moghissi et al. noted a one-year survival for 65% of the patients and 2-year survival for 47% of the patients after the intervention [12]. Among all studies that reported post-procedure degree of stenosis, the prospective case series by Gelb et al. observed more than 50% of the patients showing an endoscopic reduction in the luminal obstruction with an increase in the luminal diameter (By direct bronchoscopy estimates, the mean main-stem bronchial diameter was 1.9 mm ± 1.6 mm before laser therapy; after laser therapy, the mean diameter increased to 9.6 mm ± 1.0 mm) [13]. An increase in post-procedure FEV1, FVC, and mid-expiratory and peak expiratory flow rates was noted in the clinical investigation conducted by Gelb et al. Dyspnea index and Karnofsky score showed improvement, more for partial obstruction as compared to complete obstruction [partial obstruction: mean dyspnea index before laser (3.4 ± 0.6) and after laser (2.6 ± 0.6); complete obstruction: mean dyspnea index before laser (3.6 ± 0.5) and after laser (3.4 ± 0.5). Partial obstruction: mean Karnofsky score (%) before laser (45.0 ± 15.0) and after laser (60.0 ± 15.0), Complete obstruction: mean Karnofsky score (%) before laser (28.0 ± 11) and after laser (29.0 ± 16.0)] [16]. 

Assessment of survival outcomes showed increased median survival among patients who received multimodality treatment as compared to those who received single modality treatment (median survival after Nd:YAG laser treatment was 6.64 months; median survival in patients who received single modality treatment was 3.79 months, while median survival in pts who received multimodality treatment was 6.99 months) [1]. The maximum survival was noted to be a 47% two-year survival rate in a small study of 17 patients [17]. The procedure is deemed safe for the most part and has seldom been associated with long-term adverse effects [7,18]. The theoretical adverse effects are mostly attributed to inadvertent exposure of healthy lung tissue to the laser, which may cause tissue damage and bleeding. However, in practice, this intervention is mostly done in terminally ill patients and has shown promising palliative properties and improved survival rates. Complications of the Nd:YAG laser treatment include, but are not limited to, pneumonia, hemorrhage/hemoptysis, respiratory distress and failure, pneumothorax, tracheal-esophageal fistula, tracheal stenosis, airway rupture, arrhythmias, myocardial infarction, and death [4,6].

Nd:YAG is a well-established, safe intervention that can be offered to patients with end-stage malignancies with significant endobronchial lesions that affect the quality of life. In clinical settings, there is some evidence that bronchoscopic electrocautery might have similar outcomes in a more cost-effective manner, but more research is needed in that regard. Overall, at present, Nd:YAG continues to be a safe method to provide effective palliation in terminally ill patients [19].

Limitations

Although Nd:YAG laser has been used in clinical practice for the last four decades, there is only a limited number of prospective trials conducted to compare the effectiveness of Nd:YAG laser with that of other therapeutic and palliative measures. Most of the data available are based either on case series, case reports, or retrospective studies. The lack of standardization in the reported data makes undertaking a systematic analysis and survival analysis difficult. A number of studies reported so far lack objective data on the reduction of the size of endobronchial obstruction, improvement in the pulmonary function tests, and results on survival over an extended follow-up period. Smoking status and imaging studies are important aspects of risk assessment for survival analysis; however, a significant number of studies have failed to report these aspects in the results. Similarly, the severity of dyspnea is an important aspect to gauge the impact of treatment on the quality of life of the patients, and several studies have either failed to report it or failed to use a standard scale of assessment for the same. Given the palliative aspect and the impact on the improvement of the quality of life of the survivors reported in the studies so far, there is a need to conduct a randomized controlled prospective trial comparing the Nd:YAG laser with other different available therapeutic and palliative measures, as well as to confirm the established risk factors and objective parameters to provide objective evidence of survival benefits. 

## Conclusions

The use of Nd:YAG laser treatment in patients with luminal obstruction of the trachea-bronchial respiratory tract due to malignancy has demonstrated positive outcomes in the form of subjective and objective improvement, regardless of the age group, type of malignancy, brand of laser, degree or location of the obstruction, or the use of other modalities in conjunction to the laser treatment. Patients experienced a subjective improvement in dyspnea, hemoptysis, and cough, with some patients experiencing complete resolution of symptoms. More than 50% of the patients showed an endoscopic reduction in the luminal obstruction with an increase in the luminal diameter. Assessment of survival outcomes showed increased median survival in patients who received multimodality treatment as compared to those who received single-modality treatment. 

Based on the information currently available, Nd:YAG laser can be used as an effective form of palliative treatment to provide an improvement in symptoms and quality of life among patients with advanced-stage and inoperable endobronchial lung malignancies. There is potential for further studies that could compare median survival among patients receiving Nd:YAG laser treatment (alone or in conjunction with other modalities) in comparison to those receiving other treatments like PDT or those not receiving any treatment at all.
